# Applying user preferences to optimize the contribution of HIV self‐testing to reaching the “first 90” target of UNAIDS Fast‐track strategy: results from discrete choice experiments in Zimbabwe

**DOI:** 10.1002/jia2.25245

**Published:** 2019-03-25

**Authors:** Euphemia L Sibanda, Marc d'Elbée, Galven Maringwa, Nancy Ruhode, Mary Tumushime, Claudius Madanhire, Jason J Ong, Pitchaya Indravudh, Constancia Watadzaushe, Cheryl C Johnson, Karin Hatzold, Miriam Taegtmeyer, James R Hargreaves, Elizabeth L Corbett, Frances M Cowan, Fern Terris‐Prestholt

**Affiliations:** ^1^ Centre for Sexual Health & HIV AIDS Research (CeSHHAR) Zimbabwe Harare Zimbabwe; ^2^ Department of International Public Health Liverpool School of Tropical Medicine Liverpool United Kingdom; ^3^ Faculty of Public Health and Policy London School of Hygiene and Tropical Medicine London United Kingdom; ^4^ Faculty of Infectious and Tropical Diseases London School of Hygiene and Tropical Medicine London United Kingdom; ^5^ Malawi‐Liverpool Wellcome Trust Clinical Research Programme Blantyre Malawi; ^6^ Department of HIV/AIDS World Health Organization Geneva Switzerland; ^7^ Population Services International Johannesburg South Africa

**Keywords:** discrete choice experiments, HIV self‐testing, HIV testing, Zimbabwe, HIV, preferences

## Abstract

**Introduction:**

New HIV testing strategies are needed to reach the United Nations’ 90‐90‐90 target. HIV self‐testing (HIVST) can increase uptake, but users’ perspectives on optimal models of distribution and post‐test services are uncertain. We used discrete choice experiments (DCEs) to explore the impact of service characteristics on uptake along the testing cascade.

**Methods:**

DCEs are a quantitative survey method that present respondents with repeated choices between packages of service characteristics, and estimate relative strengths of preferences for service characteristics. From June to October 2016, we embedded DCEs within a population‐based survey following door‐to‐door HIVST distribution by community volunteers in two rural Zimbabwean districts: one DCE addressed HIVST distribution preferences; and the other preferences for linkage to confirmatory testing (LCT) following self‐testing. Using preference coefficients/utilities, we identified key drivers of uptake for each service and simulated the effect of changes of outreach and static/public clinics’ characteristics on LCT.

**Results:**

Distribution and LCT DCEs surveyed 296/329 (90.0%) and 496/594 (83.5%) participants; 81.8% and 84.9% had ever‐tested, respectively. The strongest distribution preferences were for: (1) free kits – a $1 increase in the kit price was associated with a disutility (U) of −2.017; (2) door‐to‐door kit delivery (U = +1.029) relative to collection from public/outreach clinic; (3) telephone helpline for pretest support relative to in‐person or no support (U = +0.415); (4) distributors from own/local village (U = +0.145) versus those from external communities. Participants who had never HIV tested valued phone helplines more than those previously tested. The strongest LCT preferences were: (1) immediate antiretroviral therapy (ART) availability: U = +0.614 and U = +1.052 for public and outreach clinics, respectively; (2) free services: a $1 user fee increase decreased utility at public (U = −0.381) and outreach clinics (U = −0.761); (3) proximity of clinic (U = −0.38 per hour walking). Participants reported willingness to link to either location; but never‐testers were more averse to LCT. Simulations showed the importance of availability of ART: ART unavailability at public clinics would reduce LCT by 24%.

**Conclusions:**

Free HIVST distribution by local volunteers and immediately available ART were the strongest relative preferences identified. Accommodating LCT preferences, notably ensuring efficient provision of ART, could facilitate “resistant testers” to test while maximizing uptake of post‐test services.

## 1 Introduction

HIV testing is an important entry point for uptake of prevention, treatment and care services. The United Nations 90‐90‐90 targets are that by 2020, 90% of people living with HIV should be diagnosed, of whom 90% are on treatment and 90% of those on treatment are virally suppressed 1. Although achievement of the “first 90” has already occurred in some countries, many countries have not yet attained these targets, with particularly suboptimal uptake of testing among men and young people 2, 3. HIV self‐testing (HIVST), where an individual collects his/her own oral fluid or blood sample, conducts the test and interprets results 4, is an additional testing modality that has increased the uptake and frequency of testing among individuals who would not otherwise test 5, 6. According to World Health Organization (WHO) guidelines 6, a reactive HIVST result should be followed by further confirmatory testing by a trained provider. There are several HIVST delivery models, including community‐based, workplace, public and private sector facility‐based, and secondary distribution strategies to sexual partners and peers 4.

Optimal models for distributing HIVST, which facilitate both uptake of testing and linkage to confirmatory testing (LCT), to reach those who are undiagnosed are unclear. Uncertainties around ideal service configurations include who should distribute kits, where and when they distribute them, how potential users should be engaged, and what strategies facilitate LCT. A limited number of papers have reported on preferences for service delivery characteristics that facilitate uptake of testing 7, 8 and LCT 9. Here, we report on two discrete choice experiments (DCEs) that were conducted to elicit the strength of users’ preferences for both HIVST uptake and LCT to provide recommendations on how self‐testing models can be optimized. DCEs are a quantitative survey method that elicit respondents’ preferences for attributes of goods/services/programmes 10. We also present the simulated impact of changing existing services to better support uptake of confirmatory testing.

## 2 Methods

### 2.1 Setting, model of HIVST kit distribution and support for LCT

This study is part of the Unitaid‐funded HIV Self‐Testing AfRica (STAR) project that aimed to evaluate models of distributing HIVST kits in three countries, namely Malawi, Zambia and Zimbabwe 11. In Zimbabwe, HIVST distribution was implemented by Population Services International (PSI), which conducts more than 20% of HIV tests in the country. PSI recruited and trained volunteers (community‐based distribution agents: CBDA) to distribute HIVST kits door‐to‐door. Each CBDA was a resident of the same community – a defined geographical area (all or part of a village) in which he/she distributed kits for four to six weeks. According to Ministry of Health and Child Care guidelines 12, kits were offered to all residents ≥16 years old. CBDAs each received a one‐off payment of US$50 at the end of the distribution period. To enable LCT, PSI conducted outreach visits at one and three weeks after commencement of distribution. During distribution, participants were told that they could access confirmatory testing either at PSI outreach, public clinics or any other HIV testing service. We evaluated the distribution strategy using a population‐representative survey which was conducted in one in four randomly selected households approximately eight weeks after distribution ended. We nested the distribution and LCT DCEs within the survey in two rural districts, Mazowe and Mberengwa in Mashonaland Central and Midlands provinces respectively. Participants were eligible for the survey if they were aged ≥16 years and had lived in the community for at least three months. All eligible participants in a household were recruited.

### 2.2 Defining DCE attributes and levels

To design the DCE, we used focus group discussions (FGDs) to identify key design attributes or service characteristics and levels (service options within a characteristic) that were most salient in driving decision‐making on willingness to self‐test for HIV and LCT 10. FGDs were also used to inform pictorial illustrations of attributes and their levels.

FGDs were conducted by trained social scientists; eligible participants were aged ≥16 years and had lived in the community during HIVST distribution. We based our FGD sample sizes on standard practice that would enable theoretical saturation 13. Discussions were held in the local language and were digitally recorded, transcribed and translated. Data analysis started soon after data collection began – field notes were written with view to emerging themes, followed by analytic summaries capturing both descriptive and analytic themes. These informed development of a coding framework. Coding was done using NVIVO 10.

We conducted sixteen FGDs to inform the distribution DCE (n = 150) and four FGDs for the LCT DCE (n = 33). The final attributes and levels are presented in Table 1. FGD guides and illustrations of attributes and attribute levels are presented in Appendices S1 and S2.

**Table 1:** Attributes, levels and regression coding for the HIVST distribution and LCT DCEs

Distribution DCE		LCT DCE – labelled design: Public clinic and PSI “New Start” outreach site	
Attribute	Attribute level and regression coding	Attribute	Attribute level and regression coding
Distribution method	Only directly to individuals willing to test (−1)	Proximity of clinic	Less than 30 minutes’ walk from home (0)
	Deliver tests for whole household (1)		About one hour’s walk from home (1)
Kit price	Free (0)		More than two hours’ walk from home (2)
	US$0.50 (0.5)	Busyness of clinic	Few people (−1)
	US$1 (1)		Many people (1)
Pretest support^a^	Information leaflet (−1)	Time of operation	Open weekdays 8 am to 5 pm (−1)
	Telephone helpline (1 or 0)		Open weekdays and weekends 8 am to 5 pm (1)
	Face to face from distributor (1 or 0)	Antiretroviral treatment available immediately	Yes (−1)
Time of operation	Monday to Friday 8 am to 4 pm (−1)		No (1)
	All days, including evenings and weekends (1)	User fee	None (0)
Distributor age	Below 30 years old (−1)		US $1 (1)
	Above 30 years old (1)		US $2 (2)
Distributor residence	From the same village as participant (−1)	Post‐test support^a^	None (−1)
	From outside participant village (1)		SMS reminder (1 or 0)
Location of kit collection^a^	Collection from local clinic (−1)		Call reminder (1 or 0)
	Distributed door‐to‐door (1 or 0)		In person follow‐up (1 or 0)
	Collection from mobile testing outreach sites (1 or 0)	Time between kit distribution and PSI visit (applied only to PSI outreach)	Within one week (−1)
			From two to three weeks (1)

a Since this attribute has n levels and was not treated as a continuous variable, n–1 variables indicating the level were created for that attribute. For each of these variables, where the variable takes on the omitted reference category, included categories are coded −1, otherwise the non‐reference categories take on conventional codes of 0 or 1. To retrieve the parameter for the reference category one must take: −1×sum (parameters of non‐reference categories).

### 2.3 Designing the DCE questionnaire

The DCE questionnaire, that is the specific set of repeated choices where participants choose between alternative service provision for HIVST distribution or for LCT, was generated using a d‐efficient design created in NGENE 1.0 software 14. A statistically generated experimental design ensures that the parameter or utility coefficient of each level can be retrieved with the least number of choice sets presented to the participant. DCEs assume that choices are made according to the utility maximization principle, where the best choice provides the highest utility/satisfaction to the decision maker.

For the HIVST distribution DCE, the questionnaire presented nine choice situations, each presenting two alternatives composed of seven attributes. Participants were asked to choose their preferred programme from each pair of alternatives, (Appendix S3a). For the LCT DCE, we used a design with three labelled alternatives, namely public clinic, PSI outreach testing facilities (New Start), and an opt‐out presented as “I would not confirm my reactive HIV self‐test result if these were the only two options available.” Labels are generally used when the service has multiple dimensions, which cannot be fully described, often illustrated by brand names, while the attributes and levels are objective categories that can be fully described. We considered a labelled experiment suitable for the LCT DCE as the image and status of PSI outreach versus public clinics encompasses a vast range of attitudes and preferences and are not changeable. The LCT DCE questionnaire presented twelve choice situations with three alternatives (Appendix S3b).

### 2.4 Sample size, data collection and analysis

There is no consensus on minimum sample size requirements for stated choice data 15. We employed the commonly used rule of thumb by Johnson and Orme to ensure that we were able to estimate parameters for the full sample as well as analyse preference heterogeneity between subgroups 16. We aimed to recruit 300 and 500 consecutive household survey participants in Mazowe and Mberengwa, respectively.

Paper‐based questionnaires were translated into local languages, colour‐printed and administered by trained research assistants from June to October 2016.

We estimated the parameters (utility coefficients) using discrete choice models in NLOGIT 5 software 17. All categorical attribute levels were effects coded, therefore, the parameter for the omitted level was retrieved using this formula: −1*∑coefficient of non‐omitted levels 18. According to common practice, the multinomial logistic model (MNL) was first estimated, followed by iterations of more complex models including the nested logit (NL) and the random parameter logit (RPL) to capture more complex patterns of preference heterogeneity (i.e. variation in tastes across individuals). To estimate preferences for LCT, the NL model was first tested against the MNL model because of the three‐alternative design: two LCT programmes and an opt‐out, and its relative simplicity, while allowing for some scale heterogeneity. Model fit was assessed using the Akaike information criterion (AIC); the model with the lowest AIC indicates a better statistical fit 19.

We investigated interactions with age, sex, history of HIV testing and apostolic religion. We explored age and sex since both young people and men have suboptimal uptake of testing in Zimbabwe and elsewhere in Africa 3, 20. We explored religion because the largest religious group in Zimbabwe, the Apostolic sect 21, preaches faith cure and discourages the uptake of health services 22. The above characteristics were interacted with selected attribute levels based on our literature review. All main effects (estimated on the full sample) and interaction effects (estimated by subgroups) were included simultaneously in all models.

A manual decision support system (DSS) using the nested logit model estimates was used to simulate LCT under varying service characteristics 19. Simulation was not done for the HIVST distribution DCE because we did not have an opt‐out alternative to capture a choice not to test. Simulated scenarios compared uptake of new service configurations to the base case scenario, as observed during implementation. Only attributes actionable by policy‐makers were included in the simulation exercise: approaches for supporting LCT, clinic operating time, HIV treatment availability and user fees. LCT simulations were run on the full sample and by sex and HIV testing history subgroups. We tested for statistical differences using two‐sample *t*‐tests.

Additional information on the formative qualitative phase, the DCE design, data collection and analysis methods is presented in the Data S1.

### 2.5 Ethical considerations

The study received ethical approval from Medical Research Council of Zimbabwe (MRCZ/A/2038) and London School of Hygiene & Tropical Medicine Ethics Committee (reference 11738). A written informed consent was obtained from all participants before study activities were conducted.

## 3 Results

Of 329 survey participants who were invited to participate in the distribution DCE, 296 (90%) were recruited. For the LCT DCE, an administrative challenge in the field caused a two‐day break in DCE completion by survey participants. Out of 747 survey participants seen when DCE recruitment was open, 594 were offered participation. Of these, 496 (83.5%) participated in the DCE. There were no differences between those not offered DCE participation and those who were offered by sex and marital status: 39.9% and 38.7% (*p *=* *0.8) were male, and 58.8% and 60.6% (*p *=* *0.7) were married, respectively, (results not shown).

Participants’ characteristics are presented in Table 2. More than half were women and a third were aged 16 to 25 years. Among distribution DCE participants, 54 (18.2%) had never tested for HIV, compared with 75 (15.1%) among LCT DCE participants. Across samples, we observed similar levels of education and marital status whereas the LCT DCE sample had higher employment rates than the distribution DCE sample (22.6% vs. 10.5%).

**Table 2:** Sample Characteristics

	Distribution DCE	Linkage DCE
Sample size	296, n (%)	496, n (%)
Sex		
Male	128 (43.2)	189 (38.1)
Female	168 (56.8)	307 (61.9)
Mean age (standard deviation)	37.10 (16.68)	38.61 (18.08)
Age groups		
16 to 25 years old	96 (32.4)	148 (29.8)
26 to 40 years old	89 (30.1)	136 (27.4)
>40 years old	111 (37.5)	211 (42.5)
Education level		
O level incomplete	192 (64.9)	312 (62.9)
At least O level completed	104 (35.1)	184 (37.1)
Participants’ religion		
Apostolic	134 (45.3)	176 (35.5)
Non‐apostolic	162 (54.7)	320 (64.5)
HIV testing experience		
Never tested	54 (18.2)	75 (15.1)
Self‐tested	136 (45.9)	260 (52.4)
Tested but never self‐tested	106 (35.8)	161 (32.5)
Marital status		
Married	194 (65.5)	297 (59.9)
Never married	64 (21.6)	113 (22.8)
Divorced/widowed/separated	38 (12.8)	86 (17.3)
Employment status‐receive regular salary		
No	265 (89.5)	384 (77.4)
Yes	31 (10.5)	112 (22.6)

DCE, discrete choice experiment.

### 3.1 Preference for distribution of kits

Table 3 reports findings from the MNL (Model 1) and RPL (Model 2), which both show similar results, providing some reassurance regarding the robustness of the analysis. Positive utilities show relative preference for the attribute level; a negative sign shows relative dislike. The AIC for the RPL model (AIC = 3260.9) is lower than the MNL model (AIC = 3488.3); therefore, we focus on the RPL model outputs.

**Table 3:** Models 1 and 2 estimation of preferences for HIVST distribution among the general population and by sex, age, HIV testing history and religion

	Model 1 (multinomial logit)		Model 2 (random parameter logit)			
Attribute (base case)^a^	β	SE	β	SE	SD	SE
Main effects			Random parameters			
Distribution method (Only directly to individuals)						
Deliver tests for whole household	0.008	0.051	0.055	0.115	0.632***	0.054
Kit price (per $1 increase)	−1.273***	0.272	−2.017***	0.400	1.577***	0.214
Pretest support (Information leaflet)						
Telephone helpline	0.290***	0.108	0.415***	0.152	0.048	0.158
Face‐to‐face from distributor	−0.131	0.088	−0.201*	0.120	0.069	0.202
Time of operation (Monday to Friday 8 am to 4 pm)						
Monday to Friday 8 am to 4 pm + evenings and weekends	−0.008	0.040	−0.032	0.059	0.036	0.130
Distributor age (below 30 years old)						
Above 30 years old	0.008	0.020	−0.016	0.036	0.258***	0.063
Distributor residence (from the same village)						
From another village	−0.116***	0.031	−0.145***	0.052	0.462***	0.061
Location kit collection (collection from local clinic)						
Distributed door‐to‐door	0.698***	0.219	1.029***	0.335	0.007	0.179
Collection from mobile testing outreach sites	−0.648***	0.199	−0.970***	0.309	0.404***	0.100

Interaction effects			Non‐random parameters			
Household distribution×Male	−0.057***	0.021	−0.078***	0.047		
Household distribution×Age	0.003**	0.001	0.004**	0.003		
Household distribution×Non‐tester	0.066*	0.037	0.102*	0.082		
Household distribution×Self‐tester	−0.080***	0.028	−0.130**	0.064		

Model fit statistics						
Number of participants	296		296			
Number of observations	2641		2641			
AIC	3488.3		3260.9			
AIC/N	1.321		1.235			

AIC, Akaike information criterion; HIVST, HIV self‐testing; SD, standard deviation; SE, standard error;.

^a^Since effects coding was applied, within each attribute, utility coefficients add up to zero, that is for two‐level attributes, the coefficient of the omitted level is the same magnitude with opposite sign. *10%, **5%, ***1% level of significance with *p* value.

The strongest relative preference was against paying for kits, where every $1 increase in price to users was associated with a disutility U = −2.017, *p *<* *0.01. Participants strongly preferred door‐to‐door delivery of kits (U = 1.029, *p *<* *0.01), over collection from public/mobile facilities (U = −0.970, *p *<* *0.01). For pretest support, participants strongly preferred the availability of a telephone helpline (U = 0.415, *p *<* *0.01) relative to face‐to‐face support from a distributor (U = −0.201, *p *<* *0.10) or an information leaflet alone (U = −0.214, *p*: not available).

There were significant differences in preferences for the mode of distribution of HIVST kits. Batch distribution (distribution to whole households) was preferred among non‐testers (U = 0.055 + 0.102 = 0.157, *p *<* *0.10) and older participants (U = 0.055 + 0.004 = 0.059 per year increment, *p *<* *0.05) while men (0.055 to 0.078 = −0.023, *p *<* *0.01) and self‐testers (U = 0.055 to 0.130 = −0.075, *p *<* *0.05) valued individual kit distribution. Conventional testers slightly preferred the batch distribution method (U = 0.055 + (−1×(0.102−0.130)) = 0.083, *p *<* *0.10).

The RPL model presents unobserved preference heterogeneity (variation in preferences not captured by the participants’ characteristics included in the analysis) as shown by a significant standard deviation of utility coefficients (right two columns in Table 3). For example, there was significant unobserved heterogeneity across individuals in the effect of price on their choices.

### 3.2 Preferences for LCT

The AIC shows that the NL has a better statistical fit (AIC = 8175.2) than the MNL (AIC = 8191.4 – not reported in this paper), but the RPL model (AIC = 7277.4) provided the best fit. The main and interaction effects estimated by the NL (Model 3) and RPL (Model 4) models are presented in Table 4.

**Table 4:** Models 3 and 4 estimation of preferences for LCT among the general population and by sex, age, HIV testing history and religion

	Model 3 (nested logit)		Model 4 (random parameter logit)			
Attribute (base case)^a^	β	SE	β	SE	SD	SE
Main effects	Random parameters					
**Public clinic**						
Proximity of clinic (per hour walking from home)	−0.222***	0.043	−0.348***	0.075	0.644***	0.077
Busyness of clinic (few people)						
Many people	−0.062	0.047	−0.017	0.083	0.101	0.193
Opening/operating hours (open weekdays 8 am to 5 pm)						
Open weekdays and weekends 8 am to 5 pm	0.065	0.046	0.091	0.082	0.285**	0.122
Treatment available immediately (yes)						
No	−0.565***	0.060	−0.614***	0.093	0.513***	0.162
User fee (per $1 increase)	−0.361***	0.075	−0.380**	0.166	1.015***	0.078
Post‐test support (none)						
SMS reminder	0.037	0.058	0.065	0.094	0.213	0.252
Call reminder	0.110*	0.060	0.129	0.097	0.415***	0.151
In person follow‐up	0.112**	0.055	0.143	0.090	0.336*	0.178
**PSI outreach**						
Proximity of clinic (per hour walking from home)	−0.301***	0.071	−0.328***	0.081	0.735***	0.077
Busyness of clinic (few people)						
Many people	−0.188***	0.069	−0.347***	0.091	0.708***	0.097
Opening/operating hours (open weekdays 8 am to 5 pm)						
Open weekdays and weekends 8 am to 5 pm	0.000	0.069	−0.034	0.086	0.254	0.187
Treatment available immediately (yes)						
No	−0.614***	0.070	−1.052***	0.120	1.664***	0.131
User fee (per $1 increase)	−0.454***	0.114	−0.761***	0.185	1.094***	0.081
Post‐test support (none)						
SMS reminder	0.054	0.084	0.054	0.097	0.413**	0.189
Call reminder	0.561***	0.172	0.654***	0.185	0.209	0.177
In person follow‐up	−0.031	0.082	0.118	0.095	0.214	0.281
Time between kit distribution and PSI visit (within one week)						
From two to three weeks	−0.084	0.057	−0.015	0.065	0.352***	0.098
Constant (PSI outreach relative to public clinic)	−0.218	0.188	0.194	0.155		

	Non‐random parameters					
Neither (not link to care, opt‐out)	−3.479***	0.256	−3.722***	0.237		
**Interaction effects**						
Public clinic						
SMS reminder×Non‐tester	0.152**	0.063	0.295***	0.103		
Neither (not link to care, opt‐out)						
Neither×Non‐tester	0.655***	0.104	0.717***	0.134		
Neither×Self‐tester	−0.239**	0.100	−0.243**	0.114		
Neither×Apostolic	0.145**	0.070	0.144**	0.090		
**Model fit statistics**						
Number of participants	496		496			
Number of observations	5940		5940			
AIC	8175.2		7277.4			
AIC/N	1.376		1.225			
IV parameter (nested logit)	0.569***	0.071				

AIC, Akaike information criterion; SD, standard deviation; SE, standard error.

^a^Since effects coding was applied, within each attribute, utility coefficients add up to zero, that is for two‐level attributes, the coefficient of the omitted level is the same magnitude with opposite sign. *10%, **5%, ***1% level of significance with *p* value.

There was no significant difference in preference between LCT at PSI outreach or the public clinic (i.e. the constant was not statistically significant between the two locations); what mattered were the specific service characteristics.

For both clinic types, lack of immediate antiretroviral treatment (ART) (public clinic: U = −0.614, *p *<* *0.01; PSI outreach: U = −1.052, *p *<* *0.01) was the biggest driver of choice. Consistent with the distribution DCE, participants were strongly averse to paying for services (public clinic: U = −0.380, *p *<* *0.05; PSI outreach: U = −0.761, *p *<* *0.01; per $ 1increase). The attribute of third relative importance for both locations was proximity to the health facility. Regarding post‐test support, call reminders were strongly preferred for PSI outreach. Although post‐test support options were generally not significant for the public clinic, no support at all was disliked at both locations (local clinic: U = −0.337; PSI outreach: U = −0.826; *p*: not available).

While the preference above informs drivers of where people choose to go for LCT, the opt‐out provides insights into loss‐to‐follow‐up. While most people showed a strong preference to link following a positive HIVST, the opt‐out was more often chosen among those who had never tested for HIV (U = −3.722 + 0.717 = −3.005, *p *<* *0.01) or identified as apostolic (U = −3.722 + 0.144 = −3.628, *p *<* *0.05). Those who had self‐tested chose the opt‐out option less often (U = −3.722 to 0.243 = −3.965, *p *<* *0.05), that is, they were were more likely to link for confirmatory testing at either location. This effect was stronger for those who had previously had a conventional HIV test (U = −3.722 + (−1×(0.717−0.243) = −4.196, *p *<* *0.05). Non‐testers had significantly different preferences in favour of receiving SMS reminders to support uptake of linkage at a public clinic (U = 0.065 + 0.295 = 0.360, *p *<* *0.01) relative to those who have previously tested.

### 3.3 Results of simulated linkage programmes compared to the base case scenario

**Figure 1:**
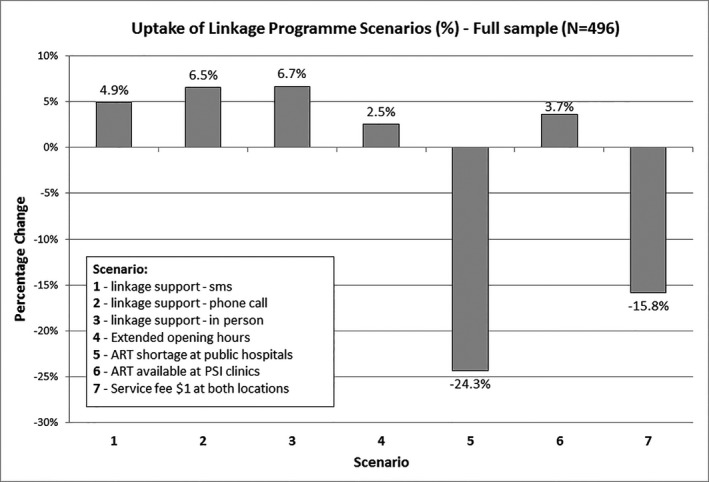
Uptake of linkage programme scenarios (%) – full sample (N = 496)

Table 5 presents a summary of the simulation exercise; Appendices S5 and S6 show full model output and simulated uptake at public clinic and PSI outreach, and Figure 1 is a graphical illustration of results of the simulation. We found that the availability of ART had the most significant effect on LCT. Shortages of ART at public clinics (scenario 5) would lead to 24.3% of respondents no longer linking. Similarly, the availability of ART at outreach facilities (scenario 6) would result in improved LCT (+3.7%) with a notable shift from public sector clinic (−6.3%) to PSI outreach (+10.0%) (Appendix S6). Introducing user fees would decrease LCT, with user fees of $1 associated with a 15.8% reduction in LCT. Analysis by sex and HIV testing history did not reveal significant differences between these sub‐groups.

**Table 5:** Change in uptake of simulated linkage programmes compared to base case for the full sample, by sex and HIV testing history (%)

Scenario	Scenario description	Full sample (n = 496), %	Female (n = 307), %	Male (n = 189), %	*t*‐test by Sex	Testers (n = 421), %	Non‐testers (n = 75), %	*t*‐test by Testing history
1	Linkage support: SMS at public clinic and PSI outreach	4.9	6.8	1.8	‐	3.5	12.4	‐
2	Linkage support: call at public clinic and PSI outreach	6.5	7.4	5.4	‐	6.9	7.8	‐
3	Linkage support: in person at public clinic and PSI outreach	6.7	7.9	4.6	‐	6.3	10.0	‐
4	Extended hours at public clinic and PSI outreach	2.5	1.6	4.0	‐	2.9	0.4	‐
5	ART shortage at public clinic	−24.3^a^	−25.0^a^	−23.6^a^	NS	−25.2^a^	−22.0^a^	NS
6	ART available at PSI outreach	3.7^a^	3.9^a^	3.1^a^	NS	3.7^a^	4.0^a^	NS
7	Service fee: $1 at public clinic and PSI outreach	−15.8^a^	−17.4^a^	−13.4^a^	NS	−16.0^a^	−15.7^a^	NS

ART, antiretroviral therapy; NS, *t*‐test not statistically significant. ^a^Significant at α = 5%.

## 4 Discussion

We found that individuals from two rural Zimbabwe districts prefer HIVST kits to be delivered door‐to‐door, free of charge and by locally based distributors. Males, young people and individuals who had already self‐tested preferred individual kit distribution rather than have kits delivered to whole households. The availability of ART was important for linkage to confirmatory testing: immediate ART initiation was most preferred while simulations showed that unstable supplies at public clinics would reduce LCT by 24.3% and introducing ART at PSI outreach would decongest public clinics as 6.3% of testers would shift to PSI outreach. People also strongly disliked payment for LCT and preferred close proximity of facilities providing confirmatory testing. Importantly, participants would rather link to either public clinic or PSI outreach than not link. Groups that were resistant to testing were also resistant to LCT. To our knowledge, this is the first paper that presents preferences related to the full HIV self‐testing cascade among participants previously exposed to community‐based HIVST.

When comparing our results with findings from other DCEs, it is important to note that differences in context typically result in exploration of different attributes. The importance of user costs is apparent: they were universally reported in three papers: one by our group reporting preference for HIVST distribution among young people in Malawi, Zambia and Zimbabwe 8, one investigating preferences for HIV testing services in Zambia 7 and the last investigating preferences for LCT following HIVST in Zambia and Malawi 9. All three reported a strong dispreference for paying for test kits or services. The DCE among young people had other similar findings that we report here, including preference for home delivery of kits by lay distributors (of note, the young people aged 16 to 25 in the distribution DCE contributed to that analysis). In contrast to our findings on preference for door‐to‐door distribution, the study that was conducted in Zambia found no significant preferences for location of HIVST distribution, although they notably did not offer participants the option for door‐to‐door delivery of kits 7. Important attributes that we report here that were not explored in other studies include immediate availability of ART and type of health facility for the LCT DCE.

Our findings show preference for the existing community‐based HIVST distribution model, with one exception: some participants wanted kits distributed to whole households (i.e. family‐based approaches). Our findings aligned with previous research; participants believed distribution to whole household would maximize testing uptake, including individuals who may not be at home during working hours 8. Also, they felt it would encourage testing among reluctant testers such as men 8. However, it was the men and young people who were opposed to household distribution of test kits, as it could potentially undermine their autonomy to decide whether they would self‐test 8. Coerced self‐testing by partners has been reported by 3% of self‐testers in Malawi, although none subsequently regretted testing 8. Incorporating distribution of kits to whole households would require concerted efforts for mitigating the potential risk of coercive testing. Men and young people have the lowest uptake of HIV testing; hence, special consideration should be given to their needs, including alternative targeted models, such as provision at workplaces, Internet and VMMC programmes.

The LCT DCE showed the importance of both immediate ART initiation and continued reliable drug stocks. This has implications for national policies relating to outreach and home‐based ART provision, which has been found to improve linkage to ART 23, and underscores the importance of ensuring reliable drug supplies. Individuals who had not previously tested preferred support through SMS reminders. This is a relatively low‐cost intervention that can be implemented to support LCT in this group, and is likely to be feasible given that Zimbabweans have good access to mobile phones 24. Notably, apostolic participants and those who had never tested for HIV were hesitant to link even if they did test, suggesting that “resistant testers” may also be “resistant linkers” for whom known status may not be enough to ensure engagement with the rest of the care cascade. In the overall survey in which the DCEs were nested, we found that 12% of participants had never tested for HIV. Interventions among this group may need to focus on shifting attitudes towards health seeking in general.

Before scale‐up of both HIVST distribution and linkage models, it is important to consider their cost and sustainability. Although the community‐based models have high impact in terms of testing groups that would not otherwise test, such as men and young people, we found that they cost more than standard provider‐delivered testing 25. Low‐cost models of ensuring door‐to‐door HIVST distribution may be important: our group is presently evaluating the feasibility and cost of community‐led HIVST distribution approaches.

The strengths of this study include use of simulations of how LCT could be affected by changes to programme attributes. We also present preferences for the full HIVST cascade. Although DCE preferences are hypothetical, our study was conducted in communities previously exposed to HIVST, so that participant preferences were shaped by their actual experiences. Using the simulation‐based RPL to account for unobserved heterogeneity improves the model fit. However, its complex structure is not well‐suited for use in simple excel‐based decision support systems, where the utilities are manually entered to predict uptake. We rather used the output from the simpler NL model to simulate the impact of variations in LCT services. Table 3 shows that although the RPL has a better statistical fit, the NL is a good approximation. Nevertheless, there are some small differences in relative utilities between the two estimators which lead to minor variations observed between the utility ranking and the simulation exercise. Another limitation is the possibility that people's preferences were shaped by current practice and experiences of self‐testing and linkage to prevention and treatment services: we did not look at how preferences varied by linkage status. Also, LCT DCE participants included those who had tested HIV negative and those who had never tested; their views could be different from those with reactive HIVST results. For the LCT DCE, labels can sometimes take away attention from other service characteristics, nevertheless, many attributes had statistically significant findings while the location was not, suggesting that choices made by participants considered the full scenario. Notwithstanding this, we did not have information on people's familiarity or use of post‐test services, which has potential to influence the choice of location of LCT services. Data were collected from only two districts, which may not be generalizable, although we do not expect that other Zimbabwe rural communities will be significantly different. Lastly, as is common with hypothetical choices, there may be a higher report of willingness to test and link.

## 5 Conclusions

We found practical insights into how HIVST could be optimized, including the needs of specific population groups such as non‐testers and those following the apostolic religion. Individuals who have resisted testing may also be resistant to linkage to confirmatory testing. Importantly, efficient provision of ART is central to engagement in post‐test services. This study contributes clients’ perspectives on how best to scale up HIVST services.

## Competing interests

No competing interests are declared.

## Authors’ contributions

ELS, FMC, FTP, MDE, ELC, MT, MT and KH formulated the research study and design. NR, MT, CM and CW collected the data and informed the design of data collection methods. MDE, GM, FTP and PI analysed the data or contributed to the analysis. ELS and MDE wrote the first draft of the manuscript. ELC, FMC, CJ, JJO, KH, JRH and FTP substantially provided intellectual input to the manuscript.

## Supporting information


**Data S1.** Design of discrete choice experiments for preferences of HIVST distribution and linkage to confirmatory testing in Zimbabwe.

**Appendix S1.** Focus Group Discussion (FGD) guides – HIVST distribution and LCT DCE.
**Appendix S2.** Attributes, levels and pictorial illustrations for the HIVST distribution and LCT DCE.
**Appendix S3a.** Distribution DCE questionnaire – Sample of one choice situation (image file).
**Appendix S3b.** LCT DCE questionnaire – Sample of one choice situation (image file).
**Appendix S4.** Selected participants’ characteristics – Spearman correlation matrices at significance level 5% (*).
**Appendix S5.** Nested logit models on the LCT DCE for the simulations among the full sample, men, women, testers and non‐testers.
**Appendix S6.** Change in uptake of simulated linkage programmes compared to base case (%) differentiated by testing facility, sex and HIV testing history.
