# Supplementary material for: Applying user preferences to optimize the contribution of HIV self‐testing to reaching the “first 90” target of UNAIDS Fast‐track strategy: results from discrete choice experiments in Zimbabwe
Source: J Int AIDS Soc. 2019 Mar 25;22(Suppl Suppl 1):e25245. doi: 10.1002/jia2.25245 (PMC6432101; doi:10.1002/jia2.25245)
Supplement: Supplementary file 1 — Data S1. Design of discrete choice experiments for preferences of HIVST distribution and linkage to confirmatory testing in Zimbabwe. [file JIA2-22-e25245-s001.docx]

**Supplementary material – Design of discrete choice experiments for preferences of HIVST distribution and linkage to confirmatory testing in Zimbabwe**

*Setting*

The DCE were conducted in two rural Zimbabwean districts, Mazowe and Mberengwa in Mashonaland Central and Midlands provinces respectively, where community-based distribution of oral-fluid test kits had recently been conducted in four and six wards respectively. [A ward is a geographic region that is designated by local government, with average adult population of about 2,500]. Residents in both communities were generally small-scale farmers. In Mazowe we explored preferences for distribution of test kits while in Mberengwa we explored preferences for LCT. Community-based HIVST distribution started in Mazowe districts; DCE results from Mazowe were aimed at informing further distribution.

*Formative qualitative phase – supplementary material*

To inform the distribution DCE, sixteen focus group discussions (FGD, total n=150 participants) were conducted following initial HIVST distribution in Mazowe district. Participants were recruited with help of CBDA who contacted them at home to invite them to participate. FGD were conducted at convenient locations within the community. Participants were asked about their views on how HIVST kits should be distributed, according to a discussion guide (Appendix 5). For the final six FGD, a ranking exercise was conducted: participants first individually ranked the importance of a pre-final list of potential attributes to be tested (based on initial FGD findings and literature search results), followed by discussions to reach consensus. Individual rankings were aggregated across participant groups to find the most important attributes, and we used discussions around group rankings to inform final selections.

To inform the LCT DCE, four FGD (n=33 participants) were held with participants who had self-tested during a pilot study in a separate district that was not used for the DCE. Participants were contacted by phone to invite them to FGDs using telephone numbers they had provided for this purpose during the pilot. Discussions were held according to a discussion guide (Appendix 5) at convenient locations in the community, and explored views on linkage to post-test services. They were followed by a similar ranking exercise to that described above.

*Attributes that were tested*

For the distribution DCE we tested participants’ preferences for: 1) whether kits were distributed to whole households or only to those who expressed willingness to test; 2) price of test kits; 3) form of pre-test support; 4) distribution working hours; 5) age of distributor, 6) residence of distributor, and 7) kit distribution/collection points. The ranking exercise deemed the following attributes unimportant: gender, whether the HIVST kit was an oral fluid test or blood-based test, and qualifications/role/profession of the distributor. For the LCT DCE we tested preferences for 1) proximity of the clinic where post-test services would be obtained; 2) timing of the outreach visit (for PSI outreach only), 3) busyness of the clinic, 4) operating hours, 5) feasibility of immediate initiation of antiretroviral therapy, 6) user fees, and 7) nature of post-test support. Attributes viewed as less important and excluded from the list were: provision of HIV services in a separate, stand-alone clinic, and time taken to access services, which was viewed as capturing similar information as busyness of the clinic.

*Designing the DCE questionnaire – supplementary material*

For each DCE, a small pilot was conducted among 50 participants to obtain preliminary estimates entered in the final NGENE design to improve the statistical robustness of the choice sets generated.

The number of scenarios to administer depends on the number of attributes and levels, the estimated sample size and the DCE design (labelled or unlabelled). The LCT DCE had more levels and was a labelled design, hence the higher number of scenarios required to reach statistical robustness. Following the d-efficient design, two different questionnaires (with different choice situations) were completed for the distribution DCE while eight were completed for the LCT DCE. The use of different questionnaires helped protect participants from treating scenarios differently.

*Data collection and analysis – supplementary material*

Research assistants were recruited by the research organisation (CeSHHAR Zimbabwe) and trained on the protocol and standard operating procedures, including how to train participants to complete DCE questionnaires (1). Before completing the questionnaires, participants underwent an exercise to familiarise them with pictorial illustrations and attribute levels. Questionnaire completion proceeded when it was clear that the participant understood.

All categorical attribute levels were effects coded, which standardises the utilities around zero rather than relative to a constant as would be the case with more conventional dummy coding. As with conventional dummy variables, the regression includes n-1 categories to avoid perfect collinearity and the omitted parameter estimate is retrieved. For effects coded categorical variables, the parameter for the omitted level is then retrieved using this formula: -1*∑coefficient of non-omitted levels in the attribute, however their p-value cannot be estimated (2).

*Manual decision support system*

The base case scenario for the public clinic assumed: one hour walking distance from home, a busy clinic and ART treatment being available. For the PSI mobile outreach, the period between HIVST distribution and outreach visit was one week, outreach clinics were not busy, within less than thirty minutes walking distance and did not provide ART. Neither provided in-person, SMS or telephone support for linkage and both offered free services within regular working hours. These base scenario characteristics were selected to represent each of these services as they run in real life. Revealed data on LCT rates from the STAR household survey showed that 60% of participants linked at public clinic, 5% at PSI outreach and 35% did not link to confirmatory testing. The simulations using stated preferences (utility coefficients) were calibrated to the revealed preferences i.e. the base scenario presented above achieves the LCT percentages from the household survey. The main assumption was that most of the population in Zimbabwe would go for HIV care services at either public hospitals or PSI New Start outreach services, therefore, those opting out of LCT for these two settings would not link at all.

The variables *HIVST kit price*, *user fee* and *age* were used as continuous (e.g. utility per one USD or year increment) and assumed a linear correlation of price and age with utilities. The rationale for not using groups is that we save on degrees of freedom, and that our assumption on linearity was as reasonable as using an arbitrary cut-off. Most importantly, data analysis showed no major differences in results between these two approaches.

1. Mangham LJ, Hanson K, McPake B. How to do (or not to do)… Designing a discrete choice experiment for application in a low-income country. Health Policy Plan. 2009;24(2):151-8.

2. Bech M, Gyrd-Hansen D. Effects coding in discrete choice experiments. Health Econ. 2005;14(10):1079-83.
