# Supplementary material for: Applying user preferences to optimize the contribution of HIV self‐testing to reaching the “first 90” target of UNAIDS Fast‐track strategy: results from discrete choice experiments in Zimbabwe
Source: J Int AIDS Soc. 2019 Mar 25;22(Suppl Suppl 1):e25245. doi: 10.1002/jia2.25245 (PMC6432101; doi:10.1002/jia2.25245)
Supplement: Supplementary file 2 — Appendix S1. Focus Group Discussion (FGD) guides – HIVST distribution and LCT DCE. Appendix S2. Attributes, levels and pictorial illustrations for the HIVST distribution and LCT DCE. Appendix S3a. Distribution DCE questionnaire – Sample of one choice situation (image file). Appendix S3b. LCT DCE questionnaire – Sample of one choice situation (image file). Appendix S4. Selected participants’ characteristics – Spearman correlation matrices at significance level 5% (*). Appendix S5. Nested logit models on the LCT DCE for the simulations among the full sample, men, women, testers and non‐testers. Appendix S6. Change in uptake of simulated linkage programmes compared to base case (%) differentiated by testing facility, sex and HIV testing history. [file JIA2-22-e25245-s002.docx]

**Appendices**

Appendix 1. Focus Group Discussion (FGD) guides – HIVST distribution and LCT DCE

**FGD guide – HIVST distribution DCE**

Opening statements: Thank you for taking time to have this discussion with us. As you may remember, my name is _____, and my colleague here is ____. Today we will have a discussion on your views on how we can detect harms that could result from HIV self-testing, which will give us important information on how we can design a good self-testing program. This discussion will take between one and a half to two hours to complete. Before we start on the questions, we would like to all agree on small rules that will help us have a fruitful discussion. (Moderator to ensure a participatory approach to setting the ground rules, which should be written on flip chart).

1. Have you ever heard of self-testing?

a. What is it and how does it differ from testing that is done by a health care worker?

b. Where did you get this information from?

c. Do you know anyone who has tested themselves for HIV?

d. What does self-testing involve?

i. Procedures

1. Processes for sample collection, analysis, and interpretation of results

2. What to do after self-testing

a. If results are negative

b. If results are positive

2. What are your views on HIV self-testing?

a. Is it a good thing? Why?

b. Will it increase uptake of HIV testing (compared to testing that is done by health care workers)?

i. What sorts of people are likely to take up self-testing?

c. Research that we have done has shown that people can test themselves and produce accurate results. What do you think about this? Do you agree that people can accurately test themselves for HIV? Why?

d. What are your views on how well people will take up prevention and care services after self-testing? Do you think people will link as well, or better, or worse than if they were tested by health care workers?

i. Uptake of medical male circumcision for HIV negative males

ii. Uptake of HIV treatment/care for those who test HIV positive

e. What are your views on social harms due to self-testing?

i. Do you think self-testing will have higher rates of social harms compared to provider-delivered testing? What sorts of harms do you think could result?

1. Forced testing? Is this likely?

a. In what types of relationship is it likely?

i. How likely is it among couples

ii. How likely is it among family e.g. parent forcing child or brother forcing sister

iii. How likely is it in the workplace e.g. employer forcing employee? Domestic helpers?

iv. Religious relationships

v. Community leadership

vi. Any other relationship types?

b. In what type of communities is it likely?

i. General communities?

ii. Institutions e.g. schools, colleges, churches?

iii. Other communities?

2. Gender based violence?

a. How likely is it for each of the above relationship types?

b. How likely is it for each of the above communities?

3. Aside from forced testing and gender based violence, are there other harms that could result from self-testing?

a. Would victims of such harms feel able to report 1) forced testing; 2) gender based violence, 3) other harms?

3. In the past few weeks, community based distributors have been in your community distributing HIV self-test kits in households. What are your views on community-based distribution of self-test kits?

a. Probe: Individual and community feelings about community-based distribution

i. What is good about community-based distribution of kits?

ii. What is not so good about it?

b. What are your views on whether people who were offered self-test kits accepted them?

i. If there is view that some people did not accept the kits:

ii. What sorts of people accepted the kits?

iii. What sorts of people did not accept them?

1. What were the reasons for not accepting the kits?

c. Do you feel the distribution that happened in your community ensured equitable access to the self-test kits?

i. Probe: Are there any sorts of people who were at a greater advantage in terms of access to kits?

ii. Are there any sorts of people who were at a disadvantage in terms of access to kits?

iii. Do you think there are people who wanted self-test kits but did not get them? Please explain

d. Do you think there are people who were given test kits yet they did not want them? Please explain.

i. What may have caused someone to take a test kit if they did not want it?

ii. What sorts of people were more likely to take kits that they did not want?

e. What are your views on whether people who got self-test kits used them?

i. If there is view that some people did not use the kits:

ii. What sorts of people used their kits? What sorts of people did not?

iii. What happened to the kits that were not used?

f. Who should distribute HIV self-test kits?

i. Existing community health workers (vanaMbuya/sekuru utsanana)?

ii. Kit distributors who were specifically appointed for the study – CBDs

iii. Others?

g. What are the preferred characteristics of a person who distributes self-test kits in the community?

i. Is age important? Why?

ii. Is gender important? Why?

iii. Is there preference on where he/she lives?

1. Within your community

2. From outside your community

h. Should distributors leave a kit for every member of the household (even those who are not home at the time of distribution) or should distribution be made only to a person who is physically present and expresses willingness to test? Please explain.

i. Are there precautions that a CBD must take when they are approaching a household to offer test kits?

i. Who to speak to

ii. Anything they should avoid saying or doing?

j. What are your views on what sorts of people should be offered self-test kits?

i. Any age restrictions?

k. How do you suggest that self-test kits be distributed?

i. The same system of using community-based distributors

1. Should they come to people’s homes or should those who want kits go to theirs?

ii. Collection from clinics

iii. Buying from pharmacies or other establishments

1. How much would people be willing to pay?

4. Before one begins the self-testing process, what are your suggestions about how he/she should be educated about the process?

5. What sort of support do you think is important before and after self-testing?

6. If self-testing were to be provided widely,

a. Would you be supportive of it? Why?

b. Would communities be supportive of it?

i. What can be done to maximise support/acceptance from the community?

7. It is possible that self-testing could result in social harms such as forced testing and gender based violence. If this were to happen, it would likely happen in secret and would be difficult to detect.

a. How could we detect episodes of forced testing in communities?

i. How could we detect forced testing in the following types of relationship

1. Forced testing between couples

2. Forced testing in families e.g. parent forcing child or brother forcing sister

3. Forced testing in employer/employee relationships

4. Forced testing in other types of relationship that were discussed earlier

ii. How would we detect episodes of gender based violence in each of the types of relationship that we have discussed?

iii. How would we detect episodes of other harms (discuss other harms aside from forced testing and GBV that participants mentioned)

8. How could we prevent these harms from occurring in our communities

a. Forced testing

b. Gender based violence

c. Other harms

9. If you could design a new service for HIV self-testing in your community, what are the components that you feel would be important to include in order prevent or minimise chances of forced testing, gender based violence or other harms?

10. The self-test kits that were distributed in your community make use of oral fluids for testing. It is also possible to do self-testing using blood, where one can do a finger prick, collect their own small sample of blood and test themselves for HIV. Which one do you think is better, using oral fluids or a blood based test?

a. Advantages of using oral fluids

b. Disadvantages of using oral fluids

c. Advantages of using a blood-based test

d. Disadvantages of using a blood based test

What do you think would be your community’s preference?

11. Do you have any questions or are there other things which are related to this topic that you would like to talk about?

**FGD guide – LCT DCE**

**General HIV testing questions**

- Aside from HIV self-testing that was recently offered to you, what current options for HIV testing in your community are you aware of?
- How are these HIV testing options viewed in the community?

Probes:

- 1. Review each listed option & briefly discuss views on the advantages and disadvantages of the service [focus on access – location, transportation, quality of staff & treatment of clients, cost of services, ability to influence service provision through complaints system/feedback]
- In general what do you think are the main reasons why people choose to go for HIV testing in your community?
- In general what do you think are the main reasons why people don’t go for HIV testing in your community?

Probes:

Mean to but just don’t get round to it?

Barriers to access including time & opportunity costs

Reluctance of individuals to acknowledge risk

Not knowing how to include their partner in the decision to test (or leave for below?)

Fear of stigma, discrimination & violence

Confidentiality & trust in service providers

Provider-client interactions (how users are treated)

Personal relationships between providers and clients

- How often, in your opinion, should Zimbabweans in general be testing for HIV? Why?
- Under what circumstances, if any, should this level of frequency of testing be different? Why?

Probes:

1. Relationship circumstances (e.g. new vs. established relationships)
2. Environment circumstances (e.g. high risk locations)
3. Occupational circumstances
4. Gender or age (e.g. male vs. female)

**Potential for self-testing**

- If you could choose to design a new service for HIV testing in your community, what are the components that you feel would be important to include?

Probes:

1. Level of supervision
2. Role of counsellors
3. Type of testing
4. Control of testing environment
5. Issues surrounding confidentiality
6. Issues surrounding accessibility

- Would you like self-testing to be regularly available to you?
- Do you think people in the community would find self-testing acceptable? (Why/why not)
- What specific conditions do you think would need to be in place in order to introduce self- testing in your community?
- If self-testing becomes available in your community, how do you think people should be able to access the self-test kits?

Probes:

1. Who should distribute self-test kits in the community
2. What role/linkages should there be with formal health services (health centres/community health workers/counsellors/VCT centres/referral services)

- if self-testing becomes available in your community, what are your views on the level of supervision that would be required to ensure it was conducted properly
- Do you think that the level of supervision should be the same for everybody choosing to self-test or should this differ according to different types of people? How?
- If self-testing becomes available in your community, who should this be targeted at individuals or couples? Should there be any age restrictions e.g. should the kits be distributed in high schools or universities? Why?

How important do you feel counselling is in currently available HIV testing services?

**The self-test kit**

- What did you think of the self-test kit in general?

Probes

- 1. Clarity of instructions
  2. Clarity of reading results
  3. Packaging
  4. Presentation and user friendliness in general
- What in your opinion are the potential advantages and disadvantages of self-testing using this test kit if it was introduced into the community?
- If it was not provided for free, how much would you be prepared to pay for a self-test kit?
- Please indicate how many of you would be willing to test yourselves again using this self-test kit? Why or why not?
- What are the most important differences between self-testing and having ordinary VCT at a facility?
- What are the most important differences that make it easier to test at home compared to testing at a facility?

**Self-testing and counselling**

- If self-testing becomes available in your community, what kind of role do you think there would need to be for counselling?
- What kind of counselling do you think might be possible in the context of self-testing?

Probes:

Telephone counselling

Referral for counselling

No counselling

Locally available community-based counselling with neighbours/strangers

- What, if any, differences should there be for counselling strategies amongst different types of people in the community?

**Self-testing and linkage to care**

- If self-testing becomes available in your community, what would be needed to help people to link to support and care services after they self-tested, for example if they tested positive?

Probes:

Information on where to go provided with the test kit

A help line number in order to call and ask where to go

A telephone call from a counsellor to help them to understand where to go

A home visit from a clinician to provide post-test services in the home

Post-test services made available locally in the community

Other ideas about how to help people link to services after self-testing?

- What, if any, differences should there be for strategies to link people to care amongst different types of people in the community?

**Safety concerns around self-testing**

- If self-testing were available in your community, how do you think people would self-test?

Probe:

Alone

With a partner or friend

At home

elsewhere

- Do you think, in general, people who self-test will tell someone about their test result?
- Did you hear of anyone giving the self-test kit to someone else to use, rather than using it themselves?
- What concerns would you have if self-testing was made available in your community?
- Do you think people who self-test will be prepared for the results?
- What could be done to help prepare people for, or help them cope with, their self-test result?
- Did you hear of anyone forcing someone else to self-test?
- If self-testing was available in your community, do you think forcing people to self-test might be a concern, for example a partner or employer? If so, how do you think this could be prevented?

Appendix 2. Attributes, levels and pictorial illustrations for the HIVST distribution and LCT DCE

| Distribution DCE | | |  | Linkage DCE - labelled design: Public clinic and PSI “New Start” outreach site | | |
| --- | --- | --- | --- | --- | --- | --- |
| **Attribute** | **Levels** |  |  | **Attribute** | **Levels** |  |
| Distribution method | *Deliver tests for whole household* | 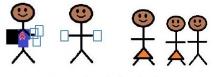 |  | Proximity from clinic | *Less than 30 minutes’ walk from home* | **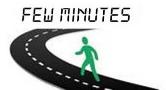** |
|  | *Only directly to individuals willing to test* | 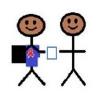 |  |  | *About 1 hours’ walk from home* | 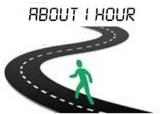 |
| Kit price | *Free* | 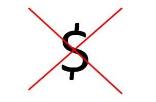 |  |  | *More than two hours’ walk from home* | 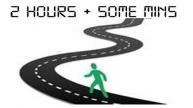 |
|  | *US$0.50* | 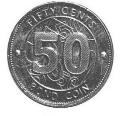 |  | Time between kit distribution and PSI visit (applied only to PSI outreach) | *Within 1 week* | *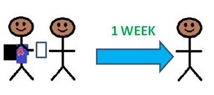* |
|  | *US$1* | 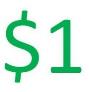 |  |  | *From 2-3 weeks* | *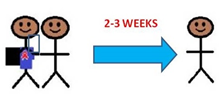* |
| Pre-test support | *Information leaflet* | 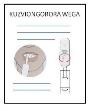 |  | Busyness of clinic | *Few people* | *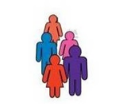* |
|  | *Telephone helpline* | 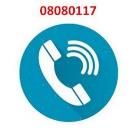 |  |  | *Many people* | **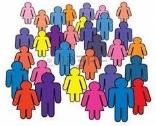** |
|  | *Face to face from distributor* | 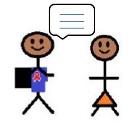 |  | Time of operation | *Open weekdays 8am-5pm* | **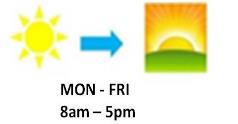** |
| Time of operation | *Monday to Friday 8am -4pm* | 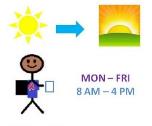 |  |  | *Open weekdays and weekends 8am – 5pm* | **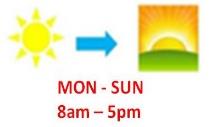** |
|  | *All days, including evenings and weekends* | 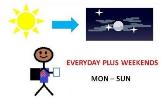 |  | Antiretroviral treatment available immediately | *Yes* | **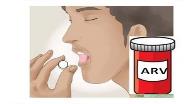** |
| Distributor age | *Below 30 years old* | 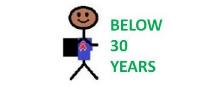 |  |  | *No* | **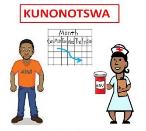** |
|  | *Above 30 years old* | 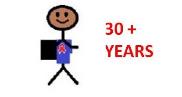 |  | User fee | *None* | **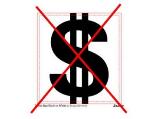** |
| Distributor residence | *From the same village as participant* | 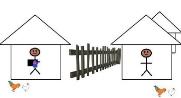 |  |  | *$1* | **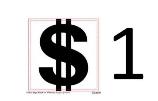** |
|  | *From outside participant village* | 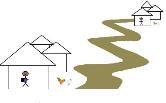 |  |  | *$2* | **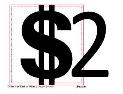** |
| Location of kit collection | *Distributed door-to-door* | 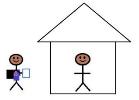 |  | Post-test support | *None* | **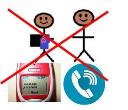** |
|  | *Collection from mobile testing outreach sites* | 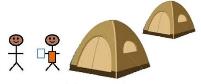 |  |  | *SMS reminder* | **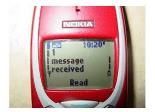** |
|  | *Collection from local clinic* | 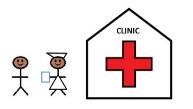 |  |  | *Call reminder* | **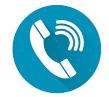** |
|  |  |  |  |  | *In person follow up* | **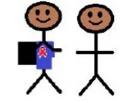** |

Appendix 3.a. Distribution DCE questionnaire – Sample of one choice situation (image file)

Appendix 3.b. LCT DCE questionnaire – Sample of one choice situation (image file)

Appendix 4. Selected participants’ characteristics - Spearman correlation matrices at significance level 5% (*).

| **Distribution DCE** | |  |  |  |  |  |  |  |  |
| --- | --- | --- | --- | --- | --- | --- | --- | --- | --- |
|  | **Age** |  | **Male** |  | **Non-tester** |  | **Self-tester** |  | **Apostolic** |
| **Age** | 1 |  |  |  |  |  |  |  |  |
| **Male** | -0.05 |  | 1 |  |  |  |  |  |  |
| **Non-tester** | -0.07 |  | 0.17 | * | 1 |  |  |  |  |
| **Self-tester** | -0.03 |  | -0.04 |  | -0.44 | * | 1 |  |  |
| **Apostolic** | 0.02 |  | -0.22 | * | -0.17 | * | 0.10 |  | 1 |
| **Linkage DCE** | |  |  |  |  |  |  |  |  |
|  | **Age** |  | **Male** |  | **Non-tester** |  | **Self-tester** |  | **Apostolic** |
| **Age** | 1 |  |  |  |  |  |  |  |  |
| **Male** | -0.07 |  | 1 |  |  |  |  |  |  |
| **Non-tester** | -0.07 |  | 0.07 |  | 1 |  |  |  |  |
| **Self-tester** | -0.05 |  | -0.03 |  | -0.44 | * | 1 |  |  |
| **Apostolic** | -0.09 | * | -0.12 | * | 0.06 |  | -0.02 |  | 1 |

Appendix 5. Nested logit models on the LCT DCE for the simulations among the full sample, men, women, testers and non-testers

|  | **Full sample (*N*=496)** | | | **Men (*N*=189)** | | | **Women (*N*=307)** | | | **Testers (*N*=421)** | | | **Non-testers (*N*=75)** | | |
| --- | --- | --- | --- | --- | --- | --- | --- | --- | --- | --- | --- | --- | --- | --- | --- |
| **Main effects** | **β** |  | **SE** | **β** |  | **SE** | **β** |  | **SE** | **β** |  | **SE** | **β** |  | **SE** |
| **PUBLIC CLINIC** | | |  |  |  |  |  |  |  |  |  |  |  |  |  |
| **Proximity of clinic** *(per hour walking from home)* | -0.216 | *** | *0.043* | -0.304 | *** | *0.07* | -0.164 | *** | *0.055* | -0.231 | *** | *0.047* | -0.139 |  | *0.101* |
| **Busyness of clinic** *(Few people)* | | | |  |  |  |  |  |  |  |  |  |  |  |  |
| *Many people* | -0.067 |  | *0.047* | -0.108 |  | *0.078* | -0.04 |  | *0.06* | -0.001 |  | *0.057* | -0.226 | ** | *0.096* |
| **Opening/operating hours** *(Open weekdays 8am-5pm)* | | | |  |  |  |  |  |  |  |  |  |  |  |  |
| *Open weekdays and weekends 8am – 5pm* | 0.061 |  | *0.046* | 0.097 |  | *0.075* | 0.04 |  | *0.058* | 0.069 |  | *0.052* | 0.011 |  | *0.096* |
| **Treatment available immediately** *(Yes)* | | | |  |  |  |  |  |  |  |  |  |  |  |  |
| *No* | -0.576 | *** | *0.06* | -0.56 | *** | *0.092* | -0.589 | *** | *0.08* | -0.598 | *** | *0.068* | -0.516 | *** | *0.125* |
| **User fee** *(per $1 increase)* | -0.632 | *** | *0.047* | -0.526 | *** | *0.077* | -0.702 | *** | *0.061* | -0.641 | *** | *0.052* | -0.621 | *** | *0.114* |
| **Post-test support** *(None)* | | |  |  |  |  |  |  |  |  |  |  |  |  |  |
| *Sms reminder* | 0.014 |  | *0.056* | -0.054 |  | *0.09* | 0.055 |  | *0.072* | -0.038 |  | *0.062* | 0.264 | * | *0.14* |
| *Call reminder* | 0.1 | * | *0.06* | 0.114 |  | *0.097* | 0.095 |  | *0.076* | 0.14 | ** | *0.066* | -0.026 |  | *0.142* |
| *In person follow up* | 0.109 | ** | *0.055* | 0.08 |  | *0.088* | 0.124 | * | *0.07* | 0.108 | * | *0.06* | 0.124 |  | *0.135* |
| **PSI OUTREACH** | | |  |  |  |  |  |  |  |  |  |  |  |  |  |
| **Constant** *(PSI outreach relative to public clinic)* | -0.218 |  | *0.188* | -0.312 |  | *0.3* | -0.157 |  | *0.24* | -0.292 |  | *0.217* | 0.034 |  | *0.467* |
| **Proximity of clinic** *(per hour walking from home)* | -0.292 | *** | *0.07* | -0.2 | * | *0.112* | -0.351 | *** | *0.09* | -0.308 | *** | *0.083* | -0.248 |  | *0.152* |
| **Time between kit distribution and PSI visit** *(Within 1 week)* | | | |  |  |  |  |  |  |  |  |  |  |  |  |
| *From 2-3 weeks* | -0.097 | * | *0.057* | -0.087 |  | *0.088* | -0.102 |  | *0.074* | -0.064 |  | *0.065* | -0.235 | * | *0.132* |
| **Busyness of clinic** *(Few people)* | | | |  |  |  |  |  |  |  |  |  |  |  |  |
| *Many people* | -0.177 | *** | *0.068* | -0.177 |  | *0.111* | -0.176 | ** | *0.086* | -0.321 | *** | *0.082* | 0.225 |  | *0.139* |
| **Opening/operating hours** *(Open weekdays 8am-5pm)* | | | |  |  |  |  |  |  |  |  |  |  |  |  |
| *Open weekdays and weekends 8am – 5pm* | 0.005 |  | *0.068* | 0.014 |  | *0.112* | -0.005 |  | *0.087* | 0.009 |  | *0.08* | -0.021 |  | *0.138* |
| **Treatment available immediately** *(Yes)* | | | |  |  |  |  |  |  |  |  |  |  |  |  |
| *No* | -0.601 | *** | *0.069* | -0.528 | *** | *0.112* | -0.644 | *** | *0.088* | -0.606 | *** | *0.081* | -0.631 | *** | *0.14* |
| **User fee** *(per $1 increase)* | -0.929 | *** | *0.09* | -0.96 | *** | *0.147* | -0.908 | *** | *0.115* | -0.942 | *** | *0.107* | -1.03 | *** | *0.184* |
| **Post-test support** *(None)* | | |  |  |  |  |  |  |  |  |  |  |  |  |  |
| *Sms reminder* | 0.038 |  | *0.083* | -0.048 |  | *0.132* | 0.094 |  | *0.107* | 0.037 |  | *0.095* | 0.085 |  | *0.194* |
| *Call reminder* | 0.012 |  | *0.086* | 0.09 |  | *0.137* | -0.042 |  | *0.11* | -0.057 |  | *0.098* | 0.285 |  | *0.223* |
| *In person follow up* | -0.025 |  | *0.08* | 0.01 |  | *0.129* | -0.046 |  | *0.103* | -0.035 |  | *0.091* | -0.007 |  | *0.192* |
| **NEITHER (NOT LINK TO CARE, OPT-OUT)** | -3.766 | *** | *0.249* | -3.784 | *** | 0.402 | -3.757 | *** | 0.318 | -4.089 | *** | 0.303 | -2.908 | *** | 0.517 |
| **Model fit statistics** | |  |  |  |  |  |  |  |  |  |  |  |  |  |  |
| Number of participants | 496 |  |  | 189 |  |  | 307 |  |  | 421 |  |  | 75 |  |  |
| Number of observations | 5952 |  |  | 2268 |  |  | 3684 |  |  | 5052 |  |  | 900 |  |  |
| AIC | 8282.8 |  |  | 3217.3 |  |  | 5091 |  |  | 6835.4 |  |  | 1428.1 |  |  |
| AIC/N | 1.392 |  |  | 1.419 |  |  | 1.382 |  |  | 1.353 |  |  | 1.587 |  |  |
| IV parameter | 0.577 | *** | 0.075 | 0.587 | *** | 0.124 | 0.574 | *** | 0.097 | 0.56 | *** | 0.083 | 0.529 | *** | 0.161 |

SE = Standard Error. *10%, ** 5%, ***1% level of significance with *p* value.

Appendix 6. Change in uptake of simulated linkage programmes compared to base case (%) differentiated by testing facility, sex and HIV testing history

|  | **Full sample (n=496)** | | | **Female (n=307)** | | | **Male (n=189)** | | | **Testers (n=421)** | | | **Non-testers (n=75)** | | |
| --- | --- | --- | --- | --- | --- | --- | --- | --- | --- | --- | --- | --- | --- | --- | --- |
| **Scenario** | **Public clinic** | **PSI outreach** | **None** | **Public clinic** | **PSI outreach** | **None** | **Public clinic** | **PSI outreach** | **None** | **Public clinic** | **PSI outreach** | **None** | **Public clinic** | **PSI outreach** | **None** |
| 1 | 5.3 | -0.4 | -4.9 | 7.3 | -0.5 | -6.8 | 2.0 | -0.2 | -1.8 | 4.1 | -0.6 | -3.5 | 12.4 | 0.1 | -12.4 |
| 2 | 7.3 | -0.8 | -6.5 | 8.5 | -1.1 | -7.4 | 5.5 | -0.1 | -5.4 | 8.3 | -1.4 | -6.9 | 5.3 | 2.5 | -7.8 |
| 3 | 7.6 | -1.0 | -6.7 | 9.1 | -1.2 | -7.9 | 5.0 | -0.4 | -4.6 | 7.6 | -1.3 | -6.3 | 9.9 | 0.1 | -10.0 |
| 4 | 2.9 | -0.3 | -2.5 | 1.9 | -0.3 | -1.6 | 4.5 | -0.5 | -4.0 | 3.2 | -0.3 | -2.9 | 0.6 | -0.3 | -0.4 |
| 5 | -27.8 | 3.5 | 24.3 | -28.4 | 3.4 | 25.0 | -27.1 | 3.5 | 23.6 | -28.8 | 3.6 | 25.2 | -25.2 | 3.2 | 22.0 |
| 6 | -6.3 | 10.0 | -3.7 | -6.7 | 10.6 | -3.9 | -5.3 | 8.4 | -3.1 | -6.3 | 10.0 | -3.7 | -6.8 | 10.8 | -4.0 |
| 7 | -13.7 | -2.1 | 15.8 | -15.5 | -1.9 | 17.4 | -10.9 | -2.4 | 13.4 | -13.9 | -2.2 | 16.0 | -13.3 | -2.4 | 15.7 |

| **Scenario** | **Scenario description** |
| --- | --- |
| 1 | linkage support: SMS  at public clinic and PSI outreach |
| 2 | linkage support: call  at public clinic and PSI outreach |
| 3 | linkage support: in person  at public clinic and PSI outreach |
| 4 | extended hours  at public clinic and PSI outreach |
| 5 | ART shortage  at public clinic |
| 6 | ART available  at PSI outreach |
| 7 | Service fee: $1  at public clinic and PSI outreach |
